# Reflecting on a decade of progress: Zero Discrimination Day and the ongoing struggle against transphobia

**DOI:** 10.1002/jia2.26227

**Published:** 2024-02-29

**Authors:** Claudia P. Cortes, Omar Sued

**Affiliations:** ^1^ Faculty of Medicine University of Chile & Fundación Arriarán Santiago Chile; ^2^ Pan American Health Organization Washington DC USA

1

On 1 March 2014, the first Zero Discrimination Day was established as a call for eradicating discrimination in all forms and promoting social inclusion and tolerance. Now, 10 years later, it is time to reflect on the important progress in the HIV response but also on the continuous challenges of stigma and discrimination. The availability of self‐testing, the efficacy of pre‐exposure prophylaxis (PrEP), the expansion of effective antiretroviral therapy (ART), and the dissemination of the undetectable equals untransmittable (U = U) message have contributed to reducing new HIV cases, decreasing mortality, and even to declaring the virtual elimination of HIV in Australia. In some countries, this information helped to shape a society that is more informed and understanding, with greater empathy, and reduced discriminatory attitudes. Grassroots movements and community organizations were key in the early days of the HIV epidemic and remain critical for amplifying the health services response to end AIDS. Some of the gains in the last few decades were accomplished by enacting and enforcing legal protections against HIV‐related discrimination in various settings—including employment, healthcare, and education for people living with HIV and key populations—and emerged from the recognition of the pervasive effects of stigma and social determinants of health on HIV‐related outcomes.

However, while significant progress has been made, not everyone enjoys the right to live with dignity regardless of their appearance, sex, sexual orientation, gender identity, ethnicity, geography, profession or beliefs. Data from the Global Report Stigma Index 2.0, which collected experiences of over 30,000 people living with HIV across 25 countries between 2020 and 2023, showed that only 57% had disclosed their status to a friend or family member. Overall, 85% reported internalized stigma that impacted their ability to find love, cope with stress or maintain ART adherence, 13% said they had experienced discrimination in HIV services in the last year and 25% reported this happened in clinical services unrelated to HIV. Notably, 15% declared that their HIV status had been disclosed to others without their consent in non‐HIV healthcare settings. All these measures were even worse among transgender people [1]. Transgender people are disproportionally affected by HIV and experience the greatest discrimination. This includes combinations of exclusion from the family, education systems and employment leading to poverty, unstable housing, engagement in survival sex work, higher exposure to violence, substance abuse and limited access to healthcare due to discrimination in the healthcare sector—all of which helps to explain the lower uptake of PrEP, higher HIV prevalence (66 times higher than the general population) and worse clinical outcomes [2].

In recent years, there has been a global recognition of the imperative to institute structural changes to mitigate the negative impact of gender‐based discrimination. Argentina led the way in recognizing the right to gender identity in 2012, passing one of the most progressive laws that permits changing gender and name on national identity cards without requiring certification by a psychiatrist, doctor, or judge. Following Argentina's example, several Latin American countries, including Uruguay, Colombia, Bolivia, Ecuador, and Peru quickly enacted similar provisions. By 2019, all countries in South America had adopted similar measures, with the exceptions of Guyana and Venezuela, and had ensured access to gender‐affirming medical interventions in public health systems. These interventions significantly improve clinical outcomes, well‐being, and mental health, particularly among youth, as documented by a reduction in the odds of suicidality by over 70% [3].

However, a surge in moral conservatism, religious influence, and political opportunism has resulted in a regression in the protection of rights for transgender individuals. In the United States, the restrictions in transgender care initiated in Florida have expanded to over 35 US states [4]. Hungary started blocking applications for legal gender recognition after 2021. Iraq introduced the death penalty for same‐sex conduct and imprisonment for transgender expression. Pakistan declared the provisions of the 2018 Transgender Act as un‐Islamic. Russia banned gender identity and gender‐affirming surgeries, and dissolved marriages of transgender people. In Uganda, the President introduced the death penalty for acts considered as “aggravated homosexuality.” [5] In the UK, the TERF (Trans Exclusionary Radical Feminist) movement, which vehemently opposes inclusive language and gender‐affirming interventions in the health system, has recently gained support from the UK government, which banned the admission of transgender women into female wards in an effort to “restore common sense.” [6]

In this challenging context, the quote attributed to Hippocrates among others “To cure sometimes, to relieve often, to comfort always” remains more pertinent than ever. Culturally competent and respectful care, in addition to tailored interventions, are essential to address the unique medical and psychosocial needs of transgender individuals.

Many healthcare workers recognize advocacy for gender‐affirming care as part of their professional responsibility to improve access to care, but also as a way to denounce marginalization, support equity and endorse legal recognition of these communities [7]. Nurses, paediatricians and family doctors can significantly contribute by providing non‐judgemental care, supporting the integration of care with mental health and social services (or facilitating referral when unavailable), enhancing capacities related to the specific needs of the population, promoting interdisciplinary care in inclusive and safe spaces, encouraging family acceptance and involving communities in the response [8, 9] (Table 1).

Governments and international agencies bear the responsibility of establishing policies that address social determinants and intersectional stigma to ensure equity, dignity and health. Therefore, we welcome the World Health Organization's announcement of the development of global guidance on transgender health, supported by more than 500 organizations [10]. Additionally, the publication of USAID guidelines for improving transgender and gender‐diverse inclusion in family planning and sexual and reproductive health service delivery is a positive step [11]. Such guidance can help us uphold the teachings of Trousseau and facilitate the fulfillment of the Hippocratic oath. However, if, for any reason, someone does not want or cannot adhere to it, another piece of advice from Hippocrates is also still relevant: “First, do no harm.”

**Table 1:** What to do to improve access to healthcare for transgender and non‐binary people (adapted from Connolly and Muschialli [9])

**Ministries of Health** Establish norms and standards for providing gender‐affirmative care. Set indicators and quality monitoring protocols. Support community organizations to implement activities for ending HIV (testing, PrEP and outreach) Require training in transgender competent care for healthcare workers. Promote intersectoral dialogues with other Ministries to address multiple vulnerabilities, including gender identity, respect for the chosen name, housing and work opportunities. Promote clinical, social and implementation research for improving health outcomes.
**Professional bodies** Acknowledge the widening health disparities and barriers to care experienced by transgender and non‐binary people as a public health crisis. Codesign and reform curricula alongside transgender and non‐binary educators, students and activists, highlighting the importance of gender identity, gender‐inclusive language and the representation of transgender and non‐binary people in all fields of medical education. Advocate for health as a human right and the need to include intersectoral interventions for improve health outcomes. Ensure professional standards are explicitly inclusive of transgender and non‐binary people (e.g. empower general practitioners to be confident offering bridging prescriptions for gender‐affirming care in circumstances laid out in the national guidance). Safeguard the wellbeing of transgender and non‐binary students and doctors. Make a public commitment and enforce policy to end harmful conversion therapy. Support clinical, social and implementation research for improving health outcomes.
**Organizations providing clinical care** Provide an inclusive clinical environment with gender‐neutral facilities and ensure transgender and non‐binary people can access facilities congruent with their gender identity. Provide cultural and transgender‐competent training to healthcare workers. Implement gold‐standard sexual orientation and gender identity recording as standard across all health systems. Evaluate health inequalities experienced by transgender and non‐binary patients and service users, publish these data and take action to address any shortcomings. Provide information about gender‐affirming interventions. Upscale the provision of gender‐affirming care with the support of expert clinicians. Adopt a zero‐tolerance policy for discrimination directed at employees, patients or general public. Liaise with local organizations that advocate for gender rights. Include peer navigators and peer support resources. Collect information through surveys and social research about stigma and discrimination.
**Health providers** Make public and accessible acknowledgements of your commitment to providing equitable care. Share your personal pronouns with patients and colleagues. Engage with publicly available educational resources to refine gender‐inclusive practice. Actively reflect on unconscious bias and commit to self‐improvement. Lead by example; be an active bystander when witnessing injustice. Promote the communication with trans organizations to work together to improve the quality of life of transgender individuals. Undertake implementation science for identifying best practices and interventions.

## COMPETING INTERESTS

The authors have declared no competing interests.

## AUTHORS’ CONTRIBUTIONS

The authors collaboratively conceived the content of the paper.

## DISCLAIMER

The opinions expressed in this viewpoint are those of the authors and do not represent the official positions of the Pan American Health Organization.
